# Deep learning and manual assessment show that the absolute mitotic count does not contain prognostic information in triple negative breast cancer

**DOI:** 10.1007/s13402-019-00445-z

**Published:** 2019-04-15

**Authors:** Maschenka C. A. Balkenhol, Peter Bult, David Tellez, Willem Vreuls, Pieter C. Clahsen, Francesco Ciompi, Jeroen A. W. M. van der Laak

**Affiliations:** 1 Department of Pathology, Radboud University Medical Center, PO Box 9100, 6500 HB Nijmegen, the Netherlands; 2 Department of Pathology, Canisius Wilhelmina Hospital, Nijmegen, the Netherlands; 3 Department of Pathology, Haaglanden Medical Center, ‘s-Gravenhage, the Netherlands

**Keywords:** Triple negative breast cancer, Mitotic count, Artificial intelligence, Prognosis

## Abstract

**Purpose:**

The prognostic value of mitotic count for invasive breast cancer is firmly established. As yet, however, limited studies have been aimed at assessing mitotic counts as a prognostic factor for triple negative breast cancers (TNBC). Here, we assessed the prognostic value of absolute mitotic counts for TNBC, using both deep learning and manual procedures.

**Methods:**

A retrospective TNBC cohort (*n* = 298) was used. The absolute manual mitotic count was assessed by averaging counts from three independent observers. Deep learning was performed using a convolutional neural network on digitized H&E slides. Multivariable Cox regression models for relapse-free survival and overall survival served as baseline models. These were expanded with dichotomized mitotic counts, attempting every possible cut-off value, and evaluated by means of the c-statistic.

**Results:**

We found that per 2 mm^2^ averaged manual mitotic counts ranged from 1 to 187 (mean 37.6, SD 23.4), whereas automatic counts ranged from 1 to 269 (mean 57.6; SD 42.2). None of the cut-off values improved the models’ baseline c-statistic, for both manual and automatic assessments.

**Conclusions:**

Based on our results we conclude that the level of proliferation, as reflected by mitotic count, does not serve as a prognostic factor for TNBC. Therefore, TNBC patient management based on mitotic count should be discouraged.

## Introduction

Recent advances in machine learning have resulted in computer algorithms that are capable of analysing entirely digitized microscopic tissue sections (whole slide images; WSI). It has been shown that such algorithms can, for instance, accurately detect and delineate tumour areas in breast and colon tissue sections and detect mitotic figures in breast cancer [1–3]. Next to direct use in research and clinical practice, such algorithms are also of interest to re-assess the diagnostic/prognostic value of widely used morphological criteria. As these algorithms allow a fully automatic analysis of large numbers of tissue sections with high reproducibility, this opens up new ways to establish ‘evidence-based’ pathology. In the present study we used deep learning to evaluate the prognostic value of mitosis counting for triple negative breast cancer (TNBC).

TNBCs comprise ~15% of all breast tumours, and are characterized by absence of expression of the oestrogen receptor (ER) and the progesterone receptor (PR) and absence of overexpression of the human epidermal growth factor receptor 2 (HER2) [4]. TNBCs are known to occur at a relatively young age and to have a worse prognosis than their hormone receptor positive counterparts [5]. In routine pathology practice, every newly diagnosed invasive breast tumour will undergo histological grading [6–8]. Histological grading of breast cancer is routinely performed by means of the modified Bloom and Richardson grading system [9, 10], which comprises a three-tiered classification system. Microscopically assessed scores, expressing the severity of nuclear pleomorphism, the relative amount of tubule formation and the mitotic count in a 2 mm^2^ area are summed up and translated into an overall histological grade. The histological grade ranges from grade 1, with features similar to normal breast epithelium, to grade 3, being most deviant from normal breast epithelium.

The vast majority of TNBCs is of histological grade 3, while grade 1 TNBCs are rare [11–14]. It has been suggested, however, that histological grade does not provide prognostic information for TNBC [11, 15]. TNBC tumours display wide ranges of mitotic counts [16, 17], with most tumours showing counts that largely exceed the minimum number required for grade 3 of the modified Bloom and Richardson grading system. These tumours, therefore, often fall in the highest class for mitotic counting, not reflecting the wide variation in absolute mitotic counts in TNBC. To date, no studies have been reported assessing the prognostic value of mitotic counts in TNBC. In addition, considering the wide range of mitotic figures present in TNBC, it may be questioned whether the cut-off values of the modified Bloom and Richardson grading system are applicable to TNBC or whether better suited TNBC-specific cut-off values are available. The application of deep learning in the present study allows for a comprehensive analysis of absolute mitotic counts, even in the presence of very high densities of such cells.

Previously, we [15] developed a multivariable prognostic model for TNBC in which histological subtype was found to serve as an independent prognostic factor. In the present study, this existing multivariable model was used as a baseline to study the added prognostic value of mitotic count for TNBC. In addition to the ‘grade 3’ cut-off value of the modified Bloom and Richardson grading system, we investigated the prognostic value of a range of alternative mitotic count cut-off values. To be able to identify alternative cut-off values in the most objective manner, we performed currently used manual assessment and average counts made independently by three pathologists, as well as a counting procedure based on state-of-the-art deep learning strategies. Combined with whole slide scanning of microscopic tissue sections, deep convolutional neural networks (CNN) [18] have been shown to be highly suited for routinely performed pathological assessments, such as metastasis detection in breast cancer sentinel lymph nodes [19] and for mitosis detection [1].

## Materials and methods

### Patients and tissue selections

In a previous study, a multicentre retrospective cohort of TNBC was established using the Netherlands Comprehensive Cancer Registry (IKNL; a nationwide registry in which all malignancies in the Netherlands are registered) [15]. The cohort comprises 597 patients who were diagnosed with TNBC between the years 2006 and 2014 in the Eastern Netherlands in an academic hospital (Radboudumc, Nijmegen) or a general hospital (Canisius Wilhelmina Hospital, Nijmegen; Jeroen Bosch Hospital, ‘s-Hertogenbosch; Bernhoven Hospital, Uden; Hospital Pantein, Boxmeer). Patients with stage IV disease on initial presentation and patients who were treated with neoadjuvant therapy were excluded. For each tumour, one representative tissue block was selected based on inspection of archival tissue sections for the presence of the tumour burden and the presence of a transition from tumour to normal breast tissue (the border of the tumour, often referred to as “invasive margin”) [20]. From every selected tissue block, one new slide was cut and stained with H&E in the Radboudumc pathology department according to routine practice. All tumours underwent central histopathological revision for histological subtype and grade (MCAB, PB) using currently applicable guidelines [10, 21].

For all patients, clinical and follow up data were retrieved from the Netherlands Comprehensive Cancer Registry (overall survival; OS) and from local patient files (relapse-free survival; RFS). The interval between the date of diagnosis of TNBC via core needle biopsy or fine needle aspiration and the date of clinically and/or pathologically detected recurrence of TNBC was defined as RFS. The occurrence of hormone receptor and/or HER2 positive breast cancer was regarded as a new primary tumour and not as a recurrence. If no recurrence occurred, patients were censored at the date of last follow up. OS was defined as the interval between the date of diagnosis of TNBC and the date of death or the moment of last follow up. The REMARK guidelines for reporting tumour marker prognostic studies were followed [22] and the study was conducted according to the Standards for Reporting of Diagnostic Accuracy (STARD) guideline [23].

### Ethical approval

The requirement for ethical approval was waived by the institutional review board (case number 2015–1711) of the Radboudumc. All patient material and data were treated according to the Code of Conduct for the Use of Data in Health Research [24] and the Code of Conduct for responsible use of human tissue in the context of health research [25].

### Manual mitosis counting

Three observers (MCAB: pathology resident; WV and PCC: pathologists with special interest in breast cancer) independently assessed the absolute mitotic counts for all tumours. All observers performed exhaustive visual mitosis counting in a 2 mm^2^ area, which was selected according to the modified Bloom and Richardson grading system [9, 10]. The absolute number of mitoses was recorded, without translating into predefined classes. All observers were blinded for any clinical or pathological information, as well as for the scores of the other observers.

### Automatic mitosis counting

**Fig. 1:**
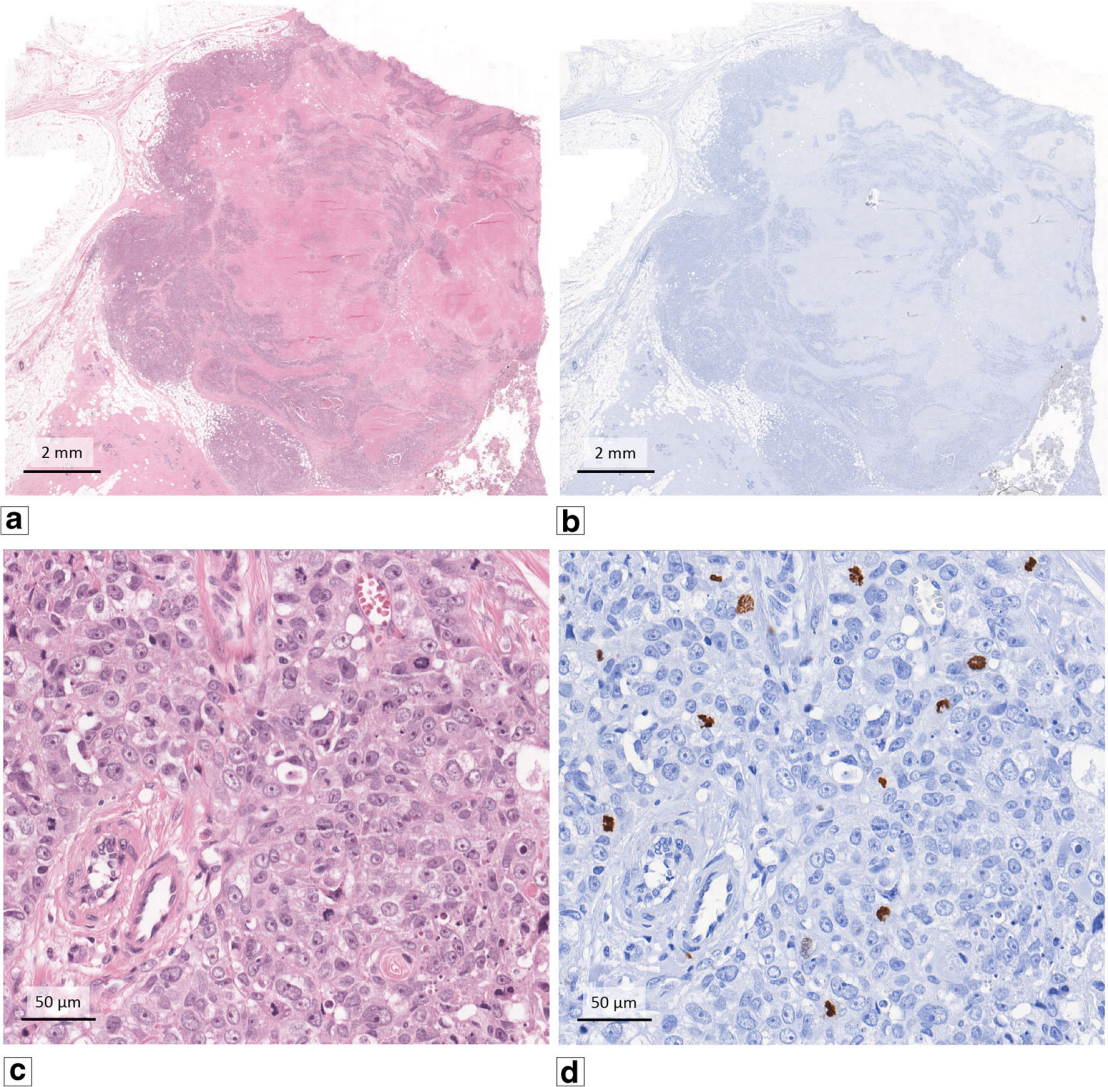
One example of the 18 TNBC cases that was used to train the deep learning algorithm. (**a**) Overview at low magnification of the additional H&E slide. After de-staining and re-staining using PHH3 (**b**), the images could be co-registered to allow precise localization of the PHH3 positive cells in the H&E section. (**c**) and (**d**) show exact correspondence between the H&E and PHH3 sections at high magnification

For automatic mitosis counting we used a previously described deep learning algorithm [1]. In brief, algorithm training was performed using 18 tumours of the TNBC cohort, for which an additional H&E slide was made and scanned on a Pannoramic 250 Flash II slide scanner (3DHistech, Hungary) at a spatial resolution of 0.25 μm/pixel. Next, slides were de-stained and immunohistochemically re-stained using an anti-phosphorylated histone H3 (PHH3) antibody, which stains mitotic figures. [26]. PHH3-stained slides were scanned using the same scanner and resolution as for the H&E slides. Resulting pairs of H&E and PHH3 whole slide images (WSI) were subsequently co-registered (i.e., images were aligned such that there was a pixel level correspondence between two images). This procedure allowed exact localization of the PHH3 positive cells in the H&E section (Fig. 1). Deep learning algorithms are typically trained using large sets of labelled examples. The set of images of mitotic figures resulting from the de-staining and re-staining procedure described above provided a very extensive training set, producing a state-of-the-art algorithm for the detection of mitoses in H&E stained slides.

**Fig. 2:**
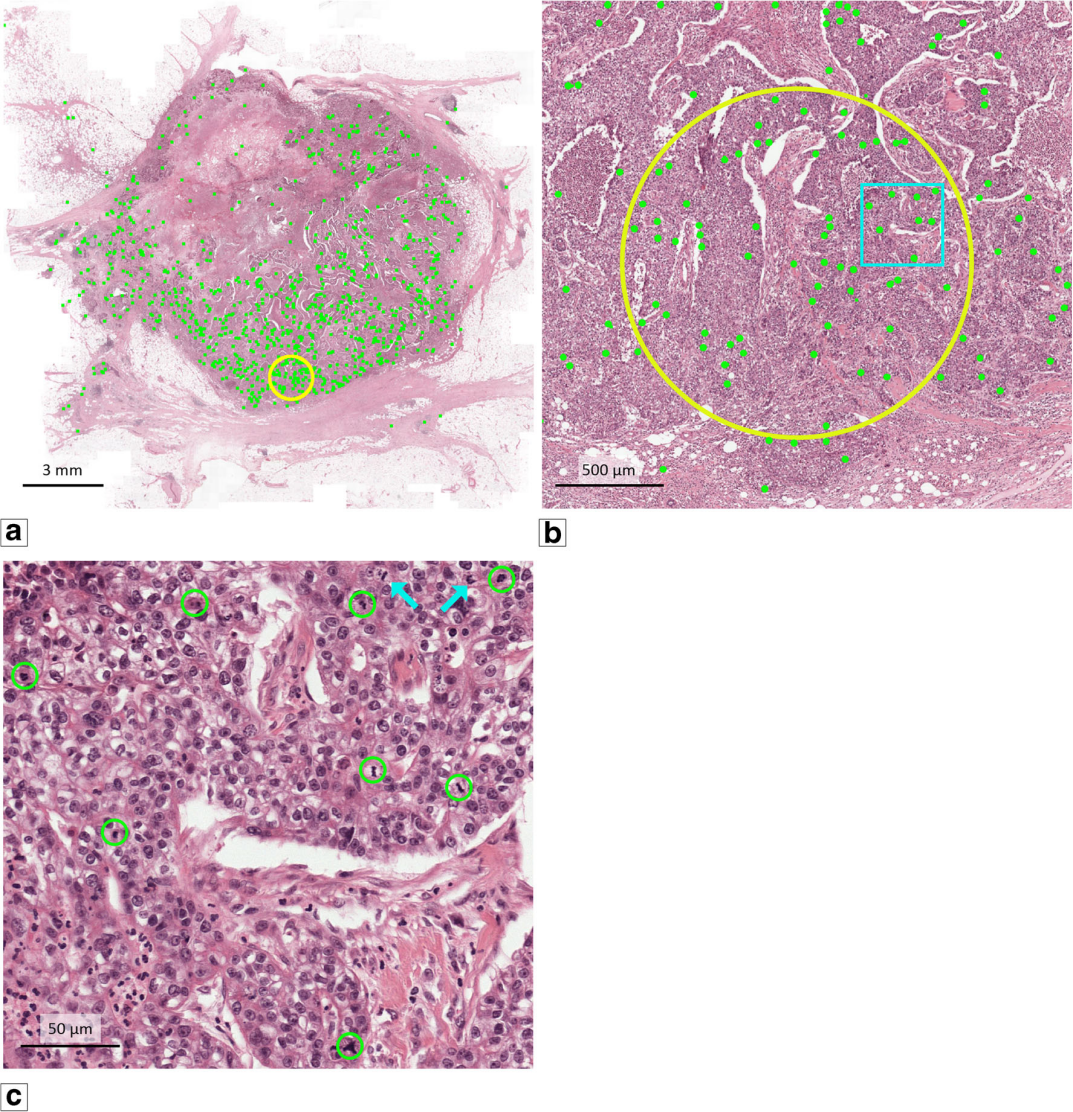
Example of the deep learning algorithm applied to one of the TNBC tumours. (**a**) Overview at low magnification of the deep learning result. Every detected mitotic figure is marked with a green dot. The yellow circle indicates the 2 mm^2^ area with the highest density of mitotic figures. (**b**) Hotspot area as found by the deep learning algorithm at higher magnification. The blue rectangle in the yellow hotspot circle relegates to the area that is shown in c. (**c**) Selected area (blue rectangle in b) at high magnification in which the mitotic figures found by the deep learning algorithm are circled in green. Two mitotic figures are missed by the algorithm (blue arrows, top right)

The H&E slides of the TNBC cohort were scanned using the same Pannoramic 250 Flash II slide scanner and settings as the algorithm was trained on. The deep learning algorithm was applied to resulting H&E WSI to automatically detect all mitotic figures (example in Fig. 2a, b). To establish an automated procedure for mitosis counting, comparable to manual counting, we calculated the number of mitoses detected by the deep learning algorithm in circles with a 2 mm^2^ area in every possible location in the WSI. Of all potential counting locations, the one with the highest mitotic density was automatically selected (Fig. 2b, c). The number of mitotic figures in this automatically identified hotspot was reported (automatic count; AMC).

For visual inspection, all automatically detected mitotic figures and the 2 mm^2^ circle were projected on the H&E WSI (Fig. 2c). Because the algorithm does not discriminate between mitotic figures in benign and malignant epithelium, a number of cases with a low mitotic density in the invasive tumour area showed a 2 mm^2^ hotspot outside the tumour (52 of the 597 tumours). In these cases, the tumours were delineated by a pathology resident (MCAB) and the algorithm was applied again, now forced to designate the hotspot in the delineated area.

### Statistical analysis

Because visual counting of mitoses is a very labour-intensive procedure, we performed a power analysis to calculate the number of tumours needed to be included for manual mitosis counting. As no literature is available on the prognostic value of the mitotic count in TNBC, we based our power calculations on studies that have reported hazard ratio’s for mitotic counts in the general breast cancer population [27–30]. The reported hazard ratio’s in these studies varied between 1.5 and > 8. For TNBC, we assume that the mitotic count yields a HR in the lower range of this spectrum. For the power analyses we, therefore, assumed a hazard ratio of 2.5. For an alpha significance level of 0.05, a power of 80% and a risk of developing a recurrence within 5 years of 20% (19.6% in the present cohort), the required sample size for a HR of 2.5 was 292. Using an overall risk of dying within 5 years of 25% (25.1% in the present cohort), the required sample size was 200. Based on these power calculations, in the present study we selected 50% of the cases (*n* = 298) of the previously described cohort [15] as follows: after ranking all patients by incidence date (date of diagnosis with TNBC by either histology or cytology) every second patient was included. The distribution of variables of interest and of number of events between the selected and un-selected cases was compared using cross tabulation. No significant differences were observed (*p* > 0.05; data not shown) using Pearson Chi-Square test. Also, independent sample T tests showed no significant differences (*p* > 0.05; data not shown) in mean time to events between selected and un-selected cases. Interobserver variability between observers and between the CNN and observers was expressed as intraclass correlation coefficients (ICC). We used a 2-way random-effects model and tested for absolute agreement with a reliability calculated from a single measure (corresponding to ICC(2,1) according to the Shrout and Fleiss convention [31]). For every tumour, the mean manual mitotic count (MMC) was calculated as the average over the three observers. Multivariable Cox regression analysis was performed to assess the prognostic value for a range of cut-off values for the MMC and the AMC, with RFS and OS as the primary outcome measures. Our previously described models [15] for RFS and OS of TNBC were used as baseline prognostic models. The baseline model consists of the variables age, primary tumour stage, regional lymph node stage, histological subtype, primary surgical treatment, adjuvant systemic therapy and adjuvant radiotherapy. Interaction terms between mitotic counts and available clinicopathological variables for RFS and OS were calculated to investigate whether the prognostic value of the mitotic count was different at different values of the other causal variable. We performed Cox analysis using the baseline model and separately adding the dichotomized MMC and AMC as a variable, for a range of different cut-off values. The lowest 10% and highest 10% of values of the MMC and the AMC were not considered as cut-off values. The range of values tested also included the cut-off value of 15, which discriminates between the classes 2 and 3 of the modified Bloom and Richardson mitotic score [10].

As a performance measure for assessment of the model, the c-statistic was used. The c-statistic indicates the discriminative power of a regression model. The approach of Harrell et al. was used to calculate the c-statistic [32], which is the preferred approach for studies focusing on long term risk prediction and in which not all individuals experience the event of interest [33]. As an additional experiment, we analysed the results of AMC on the total cohort of 597 TNBC tumours, applying the procedure described above.

For all analyses, confidence intervals were set at the 95% level and a minimal *p* value of < 0.05 was considered statistically significant. All analyses were performed using statistical software SPSS (version 24.0; IBM, Chicago, USA) and R (version 3.5.1).

## Results

### Patient demographics and tumour characteristics of the triple negative breast cancer cohort

Table 1 shows the patient and tumour characteristics of the selected cases. The majority of patients were 50 years or older at the time of diagnosis (64.8%). About half of the tumours were smaller than 2 cm (54.0%) in size. The prevailing histological subtype was invasive carcinoma NST (88.6%). Patients who were treated with chemotherapy were given anthracyclines with or without the addition of taxanes. For the group of patients treated with taxanes, no survival benefit was observed when compared to the anthracycline only patient group (data not shown). None of the patients were treated with the first generation chemotherapy regime CMF (cyclophosphamide, methotrexate, and 5-fluorouracil). In addition, no patients were treated with platinum salts. About one in five patients were confronted with a recurrence of TNBC (20.1%) and one in four patients died during the follow up period (25.5%). For the patients that developed a recurrence, the median time for developing a clinically detected recurrence was 28.8 months after primary TNBC diagnosis. The median time to TNBC-specific death was 3.7 months (mean 8.0 months) after being diagnosed with a recurrence of TNBC.

**Table 1:** Overview of patient and tumour characteristics of the triple negative breast cancer cohort (*n* = 298)

	*n*	%
Gender		
Female	298	100.0
Age, years		
≥ 50	193	64.8
< 50	105	35.2
Primary tumour stage*		
T1	161	54.0
T2	124	41.6
T3	10	3.4
T4	3	1.0
Regional lymph node stage*		
N0 (including isolated tumour cells)	206	69.1
N1	64	21.5
N2	14	4.7
N3	6	2.0
Nx (regional lymph nodes could not be assessed)	8	2.7
Histological grade [9, 10]		
1	1	0.3
2	29	9.7
3	268	89.9
Histological subtype [21]		
Invasive carcinoma of no special type	264	88.6
Special histological subtypes**	34	11.4
Primary surgical treatment		
Mastectomy	106	35.6
Breast conserving surgery	186	64.4
Adjuvant systemic therapy		
None	130	43.6
Anthracyclines	69	23.2
Anthracyclines with taxanes	97	32.6
Other regimes	2	0.7
Adjuvant radiotherapy		
No	191	64.1
Yes	107	35.9
Development of recurrence***		
No	238	79.9
Yes	60	20.1
Deceased (overall)		
No	222	74.5
Yes	76	25.5

*Primary tumour stage, regional lymph node stage and TNM stage are classified according to TNM 6th edition [34] for the years 2006 until 2009 and TNM 7th edition [35] that was in use from 2010. However, no changes considering the classification of the pathological T-stage and N-stage were made in the TNM 7th edition, resulting in comparable stages between the 6th and 7th edition

**Metaplastic carcinoma (14 patients), invasive lobular carcinoma (6 patients), adenoid cystic carcinoma (4 patients), glycogen rich clear cell carcinoma (2 patients), malignant adenomyoepithelioma (2 patients), medullary carcinoma (1 patient), invasive carcinoma with osteoclast like giant cells (1 patient)

***The presence of a recurrence was confirmed either clinically (imaging studies) or with additional pathological examination

### Baseline prognostic model

Baseline multivariable Cox regression analysis showed that a high primary tumour stage, a high regional lymph node stage and no administration of adjuvant therapy were correlated with a worse survival (Table 2). The values of the c-statistic for the baseline RFS model and the baseline OS model were 0.745 and 0.761, respectively.

**Table 2:** Baseline multivariable model for relapse-free survival and overall survival for the selected cases of the triple negative breast cancer cohort (n = 298)

	Relapse free survival HR (95% CI)	*p* value	Overall survival HR (95% CI)	*p* value
Age, years				
< 50	1 (ref)		1 (ref)	
≥ 50	0.63 (0.32–1.24)	0.179	0.72 (0.35–1.46)	0.359
Primary tumour stage*				
T1	1 (ref)		1 (ref)	
T2	1.58 (0.89–2.82)	0.121	2.85 (1.66–4.89)	< 0.001
T3	3.31 (1.23–8.94)	0.018	5.20 (1.73–15.65)	0.003
T4	NA	NA	NA	NA
Regional lymph node stage*				
N0 (including isolated tumour cells)	1 (ref)		1 (ref)	
N1	1.45 (0.72–2.91)	0.296	1.46 (0.79–2.70)	0.225
N2	9.16 (3.62–23.17)	< 0.001	4.72 (1.90–11.72)	0.001
N3	7.53 (1.99–28.41)	0.003	4.13 (0.93–18.30)	0.062
Nx (regional lymph nodes could not be assessed)	NA	NA	NA	NA
Histological type [21]				
Invasive carcinoma NST	1 (ref)		1 (ref)	
Special histological subtypes	2.21 (1.08–4.53)	0.030	1.37 (0.69–2.70)	0.369
Primary surgical treatment				
Mastectomy	1 (ref)		1 (ref)	
Breast conserving surgery	1.04 (0.54–2.00)	0.916	0.93 (0.52–1.67)	0.819
Adjuvant systemic therapy				
None	1 (ref)		1 (ref)	
Anthracyclines	0.88 (0.42–1.85)	0.730	0.32 (0.15–0.67)	0.002
Anthracyclines with taxanes	0.52 (0.23–1.19)	0.122	0.24 (0.10–0.56)	0.001
Other regimes	NA	NA	NA	NA
Adjuvant radiotherapy				
No	1 (ref)		1 (ref)	
Yes	0.56 (0.27–1.17)	0.124	0.45 (0.24–0.84)	0.012

*Abbreviations*: *CI* confidence interval, *HR* hazard ratio, *NA* not applicable, *NST* no special type, ref.: reference category

*Primary tumour stage, regional lymph node stage and TNM stage are classified according to TNM 6th edition [34] for the years 2006 until 2009 and TNM 7th edition [35] that was in use from 2010. However, no changes considering the classification of the pathological T-stage and N-stage were made in the TNM 7th edition, resulting in comparable stages between the 6th and 7th TNM edition

### Prognostic value of the mitotic count and the value of alternative cut-off values

**Fig. 3:**
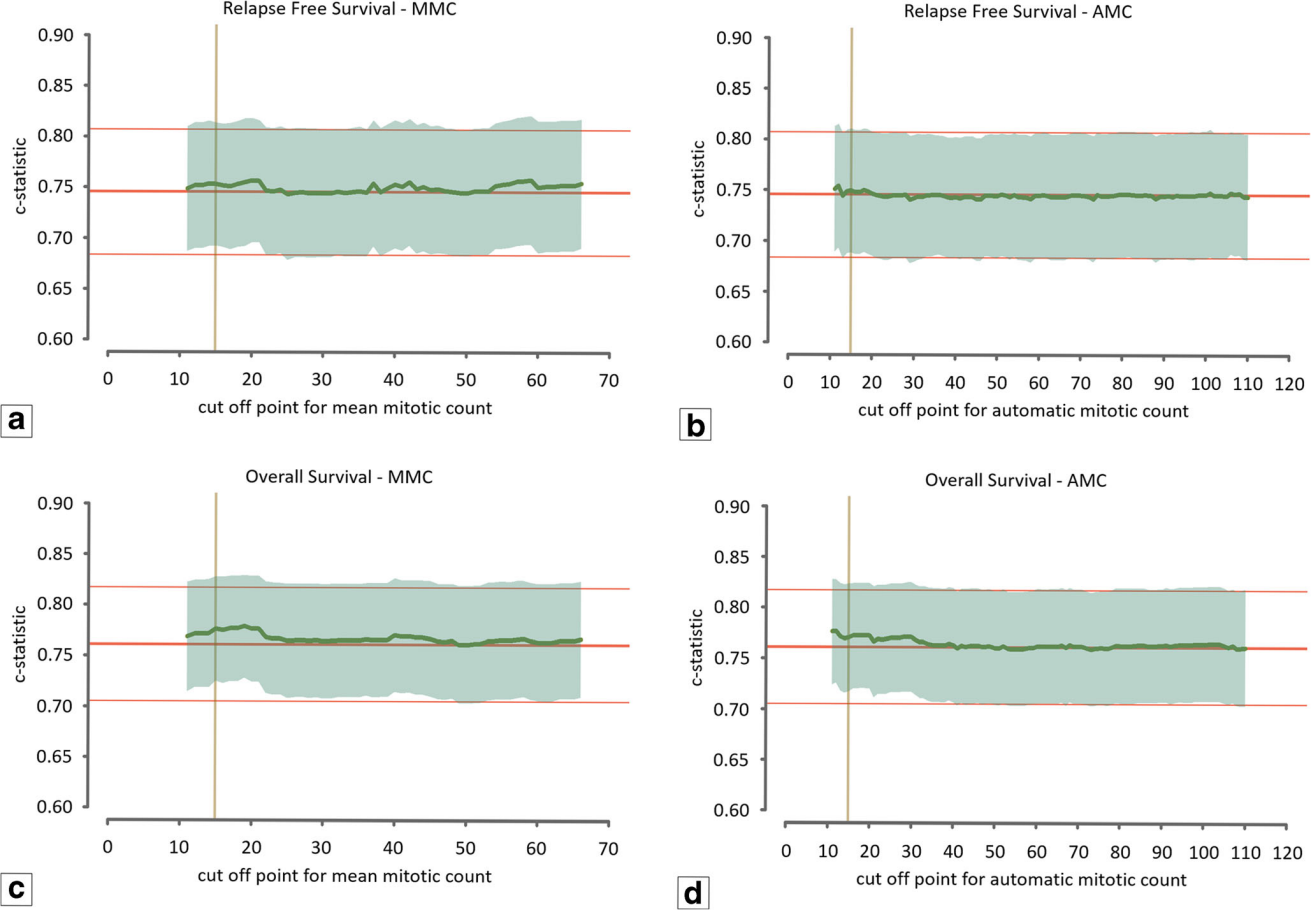
Graphic representation of the c-statistic value of the Cox regression model. The middle red line represents the value of the c-statistic of the model without the mitotic count (baseline model) with corresponding upper and lower 95% confidence interval indicated by the fine red lines. The green line indicates the value of the c-statistic for every cut-off value of the mean mitotic score for the range between 12 and 66 mitoses per 2 mm^2^ for the averaged manual assessments and between 12 and 110 mitoses per 2 mm^2^ for the automatic assessment. The translucent green area indicates the 95% upper and lower confidence interval for the value of the c-statistic. The vertical yellow line indicates the cut-off value of the modified Bloom and Richardson mitotic score (15 mitoses per 2 mm^2^). (**a**) Relapse-free survival for the mean mitotic count of the manual assessments. (**b**) Relapse-free survival for the mitotic count of the automatic assessment. (**c**) Overall survival for the mean mitotic count of the manual assessments. (**d**) Overall survival for the mitotic count of the automatic assessment

**Fig. 4:**
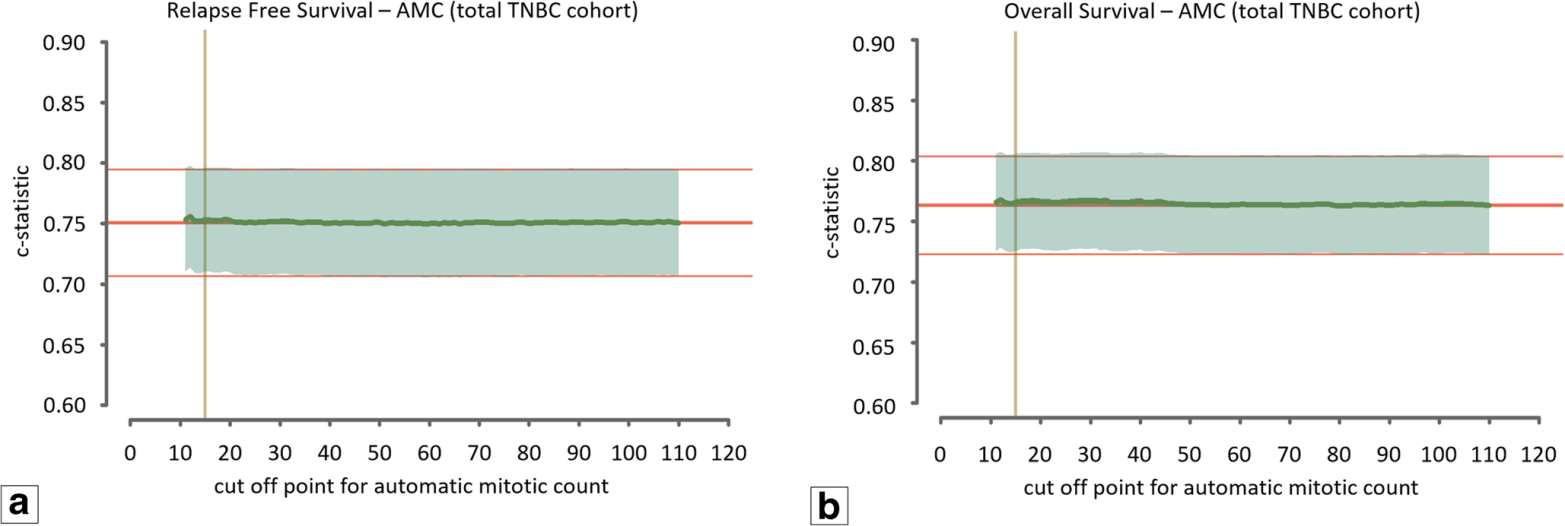
Graphic representation of the c-statistic value of the Cox regression model for the total TNBC cohort (*n* = 597) in combination with the automatic assessment of the mitotic count. The middle red line represents the value of the c-statistic of the model without the mitotic count (baseline model) with corresponding upper and lower 95% confidence interval indicated by the fine red lines. The green line indicates the value of the c-statistic for every cut-off value of the mean mitotic score for the range between 12 and 110 mitoses per 2 mm^2^. The translucent green area indicates the 95% upper and lower confidence interval for the value of the c-statistic. The vertical yellow line indicates the cut-off value of the modified Bloom and Richardson mitotic score (15 mitoses per 2 mm^2^). (**a**) Relapse-free survival. (**b**) Overall survival

The MMC ranged from 1 to 187 (median 34.7; mean 37.6; SD 23.4). The level of agreement between the three observers was good (intraclass correlation coefficient: 0.60, range 0.585–0.616). The upper and lower boundaries used for the range of cut-off values for the manual count were set at 66 and 12, respectively. The AMC ranged from 1 to 269 (median 50.5; mean 57.6; SD 42.2). The level of agreement (ICC) between AMC and observers ranged from 0.497 to 0.626. For the automatic count, the upper and lower boundaries of the cut-off values were set to 110 and 12. Figure 3 shows the discriminative capacity (expressed in the c-statistic) of the baseline prognostic model (red line) and the baseline prognostic model with the mitotic count as additional variable (green line) for both MMC (left) and AMC (right). For RFS (Fig. 3a-b), the c-statistic value of the baseline prognostic model was found to be nearly equal to the model with the addition of the mitotic count for all possible cut-off values, indicating that the mitotic count does not improve the baseline model. In addition, for OS (Fig. 3c-d) the curve that corresponds with the value of the c-statistic of the model with the addition of the mitotic count was found to fluctuate with very small margins around the curve of the c-statistic value of the baseline model. The algorithm was additionally applied to the unselected cases of the TNBC cohort using the automatic counts of the total cohort (*n* = 597) as input for the multivariable Cox regression model. Application of AMC on the extended cohort did not show any additional prognostic value of the mitotic count (Fig. 4).

### Subgroup analysis of the prognostic value of the mitotic count

**Fig. 5:**
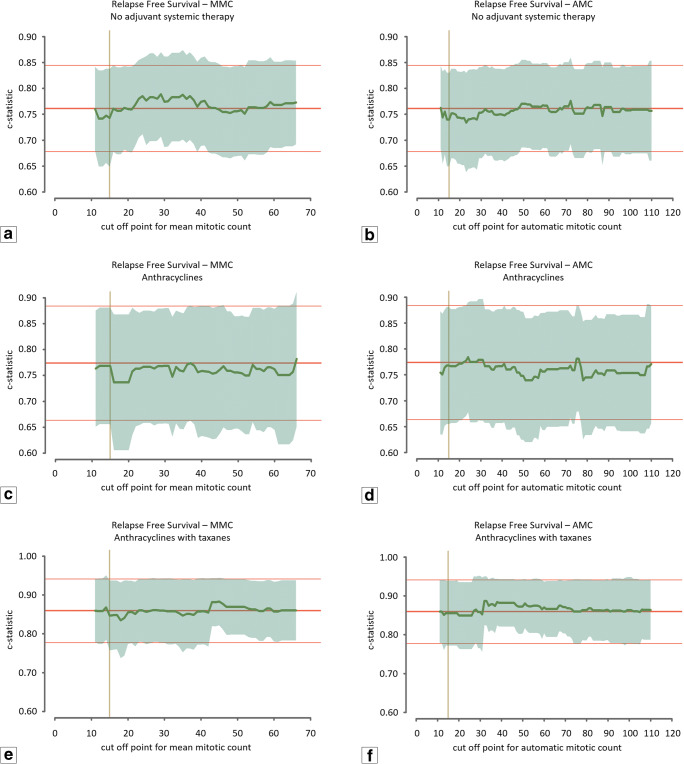
Graphic representation of the c-statistic value of the Cox regression model for the different adjuvant systemic therapy regimens; relapse-free survival. The middle red line represents the value of the c-statistic of the model without the mitotic count (baseline model) with corresponding upper and lower 95% confidence interval indicated by the fine red lines. The green line indicates the value of the c-statistic for every cut-off value of the mean mitotic score for the range between 12 and 66 mitoses per 2 mm^2^ for the averaged manual assessments and between 12 and 110 mitoses per 2 mm^2^ for the automatic assessment. The translucent green area indicates the 95% upper and lower confidence interval for the value of the c-statistic. The vertical yellow line indicates the cut-off value of the modified Bloom and Richardson mitotic score (15 mitoses per 2 mm^2^). (**a**) Mean mitotic count of the manual assessments, patients who did not receive adjuvant systemic therapy (*n* = 130). (**b**) Mitotic count of the automatic assessment, patients who did not receive adjuvant systemic therapy (*n* = 130). (**c**) Mean mitotic count of the manual assessments, patients who received anthracycline-based chemotherapy regimens (*n* = 69). (**d**) Mitotic count of the automatic assessment, patients who received anthracycline-based chemotherapy regimens (*n* = 69). (**e**) Mean mitotic count of the manual assessments, patients who received anthracycline with taxane-based chemotherapy regimens (*n* = 97). (**f**) Mitotic count of the automatic assessment, patients who received anthracycline with taxane-based chemotherapy regimens (*n* = 97)

**Fig. 6:**
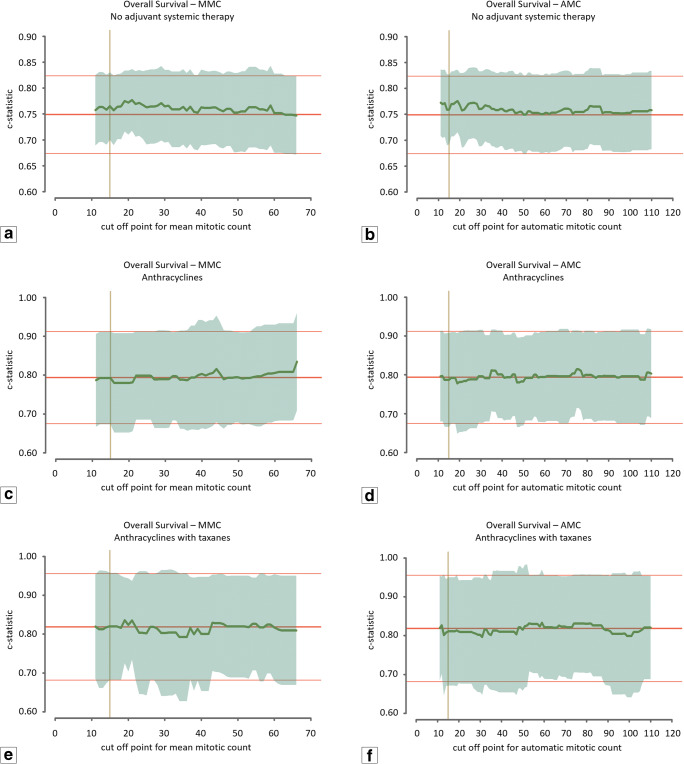
Graphic representation of the c-statistic value of the Cox regression model for the different adjuvant systemic therapy regimens; overall survival. The middle red line represents the value of the c-statistic of the model without the mitotic count (baseline model) with corresponding upper and lower 95% confidence interval indicated by the fine red lines. The green line indicates the value of the c-statistic for every cut-off value of the mean mitotic score for the range between 12 and 66 mitoses per 2 mm^2^ for the averaged manual assessments and between 12 and 110 mitoses per 2 mm^2^ for the automatic assessment. The translucent green area indicates the 95% upper and lower confidence interval for the value of the c-statistic. The vertical yellow line indicates the cut-off value of the modified Bloom and Richardson mitotic score (15 mitoses per 2 mm^2^). (**a**) Mean mitotic count of the manual assessments, patients who did not receive adjuvant systemic therapy (*n* = 130). (**b**) Mitotic count of the automatic assessment, patients who did not receive adjuvant systemic therapy (*n* = 130). (**c**) Mean mitotic count of the manual assessments, patients who received anthracycline-based chemotherapy regimens (n = 69). (**d**) Mitotic count of the automatic assessment, patients who received anthracycline-based chemotherapy regimens (*n* = 69). (**e**) Mean mitotic count of the manual assessments, patients who received anthracycline with taxane-based chemotherapy regimens (*n* = 97). (**f**) Mitotic count of the automatic assessment, patients who received anthracycline with taxane-based chemotherapy regimens (*n* = 97)

The relation between the mitotic counts and the available clinicopathological variables were calculated using interaction terms. The only variable for which interaction with the mitotic count was found was type of adjuvant systemic therapy. Figures 5 and 6 visualise the baseline c-statistics and additional prognostic value of the mitotic counts for the different adjuvant systemic therapy regimes. Due to too small numbers, no calculation for the patients who received other systemic therapy regimens (*n* = 2) could be performed. Figures 5a and 6a show that for a selection of cut-off values, the c-statistic of the model that includes the mitotic count (green line) exhibits higher values than the baseline c-statistic. No cut-off value, however, exhibited values outside the confidence intervals of the baseline models (fine red lines).

## Discussion

Previously, we showed that histological subtype is an independent prognostic feature for RFS in TNBC [15]. In the present study we explored the prognostic value of the mitotic count for TNBC. The multivariable Cox regression model developed in our prior study served as baseline model and was used to assess the putative added prognostic value of the mitotic count for TNBC. The Cox regression models for RFS and OS were expanded with the mitotic count, after which we analysed the discriminative value of the regression model for a wide range of cut-off values for the mitotic count. We found that the addition of the mitotic count did not improve the prognostic model for RFS or OS for any of the tested cut-off values.

TNBC tumours are characterized by the absence of ER and PR expression and the absence of HER2 overexpression. As such, the TNBC category contains a mixture of tumours with widely varying histopathological and genomic characteristics and, consequently, variable clinical courses of the disease. This situation poses challenges for the treating clinician, as the basis for therapy choices is small. Well-known prognostic factors are not yet established for TNBC. Recent work has focused on identifying immune-based and molecular features of TNBC to obtain prognostic [36, 37] and predictive [38–40] biomarkers. For the TNBC subtype, only scarce literature exists focusing on the prognostic value of proliferation. It has been shown that TNBC tumours express high levels of proliferative activity on the mRNA [41] and protein levels [42]. However, so far no strong proliferation-based prognosticator specifically for this type of cancer has evolved. We previously found that histopathological subtype serves as an independent prognostic factor, potentially identifying a small subgroup (up to 30% [11–13]) of TNBC with a worse prognosis. The present study shows that mitotic counting does not yield any prognostic information for TNBC. To the best of our knowledge, this is the first study that focused on the prognostic value of absolute mitotic counts for TNBC. Mitotic counting as part of tumour grading is considered an established prognostic factor for breast cancer in general, and is routinely performed for every new case. Results from the present study indicate that we may have to re-consider the current practice of grading TNBC. Clearly, alternative prognostic factors for TNBC are urgently needed.

Although many studies have assessed the prognostic value of the mitotic count for invasive breast cancer, the prognostic value of the absolute mitotic count is not commonly studied. The mitotic count is typically analysed as a categorical variable that comprises three classes (low, middle and high) with cut-off values that are based on the modified Bloom and Richardson grading system [43, 44]. An exception is the study design of Kronqvist et al. [45, 46]. The prognostic value of a range of thresholds of the mitotic count was analysed by these authors in a cohort of 364 breast cancer patients. An optimal cut-off value for the mitotic count was determined by examining Chi-square values of the log rank test for every cut-off value for different subgroups of patients. Although this approach bears similarities with our study design, the most important difference is that our current study explores the prognostic value of cut-off values specifically for TNBC, which are known to exhibit much higher mitotic counts than general breast cancers. Also, we performed a multivariable approach to correct for other prognostic factors, in contrast to the univariable analyses applied by Kronqvist et al.

It has been shown that counting of mitotic figures is prone to subjectivity [42] due to a lack of standardization [47, 48] and, additionally, is hampered by external factors such as fixation artefacts [49]. To become less dependent on individual assessments of the number of mitotic figures and of the selection of the area where to count, three observers independently assessed the mitotic count for every tumour in our study. We used the average of the three observers as the mean mitotic count for every tumour. By taking the average of the individual counts the interobserver variation was reduced. In addition, we applied a deep learning algorithm to assess the mitotic counts in an objective and reproducible manner. Automated counting based on deep learning potentially yields a more objective and reproducible measure [50]. Also, because of the extensive effort associated with manual counting of larger numbers of mitoses, only by using automated counting we could analyse the prognostic value in the entire cohort of almost 600 cases, adding considerable power to the study. The use of a deep learning-based algorithm in the present study showcases the potential of such techniques for re-evaluating existing histopathological features.

Next to research use, deep learning will most likely also find clinical application in histopathology. Algorithms such as the one used in the present study may be very useful in a clinical setting, pre-analysing scanned tissue sections before the pathologist starts the diagnostic process. With the introduction of whole slide imaging devices, the introduction of such algorithms will be strongly facilitated. An algorithm that has already processed WSI can subsequently be used to assist pathologists to reduce observer biases, and increase accuracy and efficiency. The first study on the potential of such a setup in a prospective setting showed that screening for metastases in sentinel lymph nodes of breast cancer patients was both faster and more accurate when a pathologist was assisted by a deep learning-based system [51].

This study has several strengths. Because our TNBC cohort was deduced from 5 different hospitals over several years, it can be regarded as a good reflection of the diverse TNBC population. Counting mitotic figures in H&E slides is a laborious and time-consuming task. The time and effort that the three observers have put into counting the absolute numbers of mitotic figures in nearly 300 tumours is, therefore, very valuable. The additional analyses of mitotic counts assessed by a computer-based algorithm, which showed comparable prognostic value as the counts of the human observers did, underscore the conclusion that mitotic count is of no prognostic value for TNBC. As stated, no previous reports have been published studying the prognostic value of mitotic counts in TNBC. Our analysis is limited by the constraints of a retrospective study, although we made a considerable effort to obtain high quality and complete follow up data by using a highly reliable nationwide cancer registry and an extensive review of the patient files. In conclusion, by analysing a wide range of cut-off values, we show that mitotic count does not improve the prognostic value of currently available multivariable prognostic models for RFS and OS for TNBC. Our results suggest that mitotic count is of no prognostic value for TNBC patients. As this is the first study that specifically explored the prognostic value of mitotic counts for TNBC, these results must be confirmed in independent TNBC cohorts. In addition, this study shows the potential of deep learning-based algorithms for evaluating histopathological features in large series in an objective manner.
